# Evaluation of the Health-related Behaviour of Pregnant Women from Warsaw, Poland

**Published:** 2018-01

**Authors:** Dariusz BOGUSZEWSKI, Jakub Grzegorz ADAMCZYK, Wiesław TOMASZEWSKI, Daria SAŁATA, Ewelina SKOWERA, Marta PATALON, Anna OBSZYŃSKA-LITWINIEC, Dariusz BIAŁOSZEWSKI

**Affiliations:** 1. Division of Rehabilitation, Dept. of Physiotherapy, 2nd Medical Faculty, Medical University of Warsaw, Warsaw, Poland; 2. Theory of Sport Department, Faculty of Physical Education, University of Physical Education, Warsaw, Poland; 3. College of Physiotherapy, Wroclaw, Poland

**Keywords:** Lifestyle, Pregnant women, Physical activity, Health-related behaviour

## Abstract

**Background:**

Pregnancy is a period of time when women tend to suffer from the weakening of their psychophysical fitness. This research evaluated several selected elements of the lifestyle of pregnant women compared to those of non-pregnant women.

**Methods:**

Overall, 482 women attended to the Childbirth School in Gynecological-Obstetric Hospital “Inflancka” in Warsaw, Poland, in the years 2011–2013; Group 1 contained 214 pregnant, and Group 2 contained 268 non-pregnant completed a survey inquiry. The research tool applied was Juczyński’s “Inventory of Health Behaviour” (Inwentarz Zachowań Zdrowotnych). In this tool, the author evaluates health behaviors through four separate categories: dietary habits, prophylactic behaviors, mental attitude, and health behaviors. The differences between the data were defined through the Student’s t-test for independent groups, with a minimal level of significance set at *P* ≤ 0.05.

**Results:**

Pregnant women take care of following a healthy lifestyle. The general health behaviour index figure was significantly higher in Group 1 as compared with the Group 2 (*P*<0.001). A higher level (*P*<0.001) of healthy behaviour was typical of physically-active individuals, regardless of their Group (1 & 2).

**Conclusion:**

Pregnancy might cause women to increase their interest in matters of their own health and adopt a healthier lifestyle. Physical activity can influence other health-related practices.

## Introduction

According to the National Health Programme (Narodowy Program Zdrowia), lifestyle has the most vital impact on both female health during the course of pregnancy and on the health of her future child (50%–60%). The remaining factors are as follows: both the social and natural environments of her location and employment (approx. 20%), genetic factors (approx. 20%), and health care (10%–15%). Health behaviours and lifestyle most significantly determine the level of health (1, 2).

Pregnant women’s health practices should serve the purpose of promoting an optimal course of pregnancy and condition of the mother’s health. With regard to this, pregnant women should follow a few prescriptions concerning hygiene and lifestyle (3, 4). A key factor is the type of diet followed. To assume a rational everyday diet, an appropriate volume of energy must be commensurately provided with all of the indispensable nutritional compounds (fats, protein, carbohydrates, vitamins, and minerals). Both a deficiency and superfluity of dietary ingredients can be detrimental to a pregnant woman and her developing child alike. Equally important for the proper development of the fetus is an adequate diet both prior to and during the pregnancy period. Certain vitamin and mineral deficiencies may result in adverse health consequences (5).

One of the most well-intentioned ways that a woman may care about her lifestyle, both during pregnancy and following childbirth, is through conscious physical activity. Therefore, one of the essential elements of the so-called school of childbirth provides a set of special exercises, which aim at supporting the stamina of pregnant woman and facilitating the later stages of child delivery (6–9).

For pregnant women who are able to do some level of aerobics, a set of norms has been designed to emphasize safety, energetic balance, and lowering stress levels (10). Any instructions for completing exercises during pregnancy should guarantee the improvement of both the health and quality of life of the mother. The following are suggested as exercise activities: aerobics such as cycling, jogging, fitness (of an appropriate level of intensity), and swimming, as well as breathing deeply through the diaphragm – essential for successful delivery. Exercises with a high risk of falling or injury are strictly not recommended (10–12).

The common disorders frequently experienced by pregnant women, such as varicose veins, swollen limbs, fatigue, and chronic back pain, are significantly less common in women who undergo exercise (13). Additionally, these women also experience relatively fewer disturbances to their sleep, as well as less stress, depression, and fear (14, 15). Exercises with weights done during the period of pregnancy beneficially narrow the span of delivery and reduce some potential complications that may occur after childbirth (16).

Adhering to a sedentary lifestyle during pregnancy diminishes muscle stamina and the proper performance of the circulatory system. This may result in a gain in body weight and enhances the threat of diabetes and preeclampsia (17, 18). Additionally, this lifestyle contributes to an increase in the frequency of such disorders such as varicose veins, discomfort in the spine and peripheral joints, and difficulty breathing (*dyspnoea*). It may also have a negative impact on women’s psyches, as they might not fully accept and adapt to the changes that occur in their bodies during pregnancy (14, 15).

Lifestyle plays a key role in maintaining appropriate health levels and quality of life while pregnant. After conception, women tend up to give up many forms of activity, replacing them with activities that are more passive. It is for this reason that some forms of active and healthy lifestyle promotion can minimise painful disorders and improve individual physical dexterity, with its effects eventually extending beyond pregnant women to society at large (2, 19).

The main purpose of this research was to evaluate the health behaviours of women far along in their pregnancies in comparison to non-pregnant women. An effort was made to define the factors influencing the lifestyle of pregnant women as well.

## Material and Methods

Four hundred eighty women, all inhabitants of Warsaw, Poland, attended to the Childbirth School in Gynecological-Obstetric Hospital “Inflancka” (Warsaw, Poland), were divided into two groups. Group 1 (n = 214) was made up by women far along in their pregnancy (29.81 wk of pregnancy on average, with SD = 3.42). Group 2, the pilot group (n = 268), covered non-pregnant women. The average age was 27.97 yr old (± 5.55). The study was conducted in 2011, 2012 and 2013.

The research was approved by the local Ethic Committee and all participants gave informed consent before the study.

All surveyed women were inhabitants of Warsaw. Individuals qualified for the survey were healthy and not affected by any enduring illness. Additionally, for the purpose of some comparative studies, another division was introduced, whereby sub-groups were distinguished between groups who were younger or older than thirty years old as well as physically active or non-active. The physically active sub-group included women who frequented aerobics class at least twice a week. A general biometric performance of the examined respondents is provided in Table 1.

**Table 1:** Characteristics of the groups (mean values ±SD)

	***Age (yr)***	***Body height (cm)***	***Body mass (kg)***	***BMI***
Group 1 (n=214)	28.98 ±4.32	167.41 ±5.59	69.89 ±9.28	24.94 ±3.04
Group 2 (n=268)	26.35 ±6.89	166.44 ±5.68	58.71 ±6.93	21.05 ±2.17

The research tool applied was the “Inventory of Health Behaviour” by Juczyński, where health behaviour is evaluated according to four categories: dietary habits, prophylactic behaviours, mental attitude, and health behaviours (20). The questions in the questionnaire stem from four health behaviour categories, namely nutrition habits NH – correct diet habits, types of food being consumed, prophylactic behaviour PB – observing the rules of health care, obtaining information about health and disease, positive (mental) attitude PA – avoiding debilitating emotions, strains, and stress together with healthy practices HP – daily activities comprising aerobics, sleep, and relaxation. Sum of all points gives health-related behaviours index (HRB). Additionally, an authorial inquiry was carried out to gather some bio-metric information (20).

The data were examined with the help of some standard statistical tools, comprised of an arithmetic mean together with a standard deviation. The differences between the particular sets of data were calculated through the Student’s *t*-test for independent groups. Any observed correlations amongst the qualities were defined by the Pearson coefficient. The minimal level of significance was determined at a level of *P*≤ 0.05.

## Results

With reference to the data gathered through the Inventory of Health Behaviour, the level of stated behaviour and activities linked to health in the examined groups reached an average level (the value of the index figure of HRB for Group 1 is 87.56, while for Group 2 it is 78.81). Pregnant women showed higher levels of healthy behaviour (*P*<0.001). Significantly, higher results for Group 1 were observed across all four components included in the IHB questionnaire, including dietary habits, prophylactic behaviours, positive mental attitude, and health behaviours (Table 2).

**Table 2:** Level of health-related behaviour (NH – nutrition habits, PB – prophylactic behaviour, PA – positive attitude, HP – healthy practices, HRB – health-related behaviours)

***Item***	***NH***	***PB***	***PA***	***HP***	***HRB***
Group 1 (n=214)	3.66 ±0.68	3.56 ±0.71	3.69 ±0.68	3.68 ±0.67	87.56 ±12.59
Group 2 (n=268)	3.33 ±0.77	3.2 ±0.74	3.43 ±0.71	3.18 ±0.67	78.81 ±12.37
Difference (*P*)	*P*<0.001	*P*<0.001	*P*<0.001	*P*<0.001	*P*<0.001

Taking into account the division into sub-groups, women under 30 yr old manifested substantially higher levels of healthy behaviour in all categories. Minor differences were recorded among individuals above 30 yr old. Essentially, higher levels of healthy behaviour were observed in those regularly doing aerobics in both groups (Table 3).

**Table 3:** Level of health-related behaviour in subgroups (NH – nutrition habits, PB – prophylactic behaviour, PA – positive attitude, HP – healthy practices, HRB – health-related behaviours)

	***Criteria***	***Group***	***NH***	***PB***	***PA***	***HP***	***HRBI***
Age (yr)	<30	Group 1 (n=130)	3.61 ±0.68	3.54 ±0.74	3.73 ±0.69	3.67 ±0.69	87.28 ±12.62
		Group 2 (n=209)	3.29 ±0.72	3.12 ±0.71	3.38 ±0.64	3.15 ±0.63	77.65 ±11.49
	Difference (p)		*P*<0.001	*P*<0.001	*P*<0.001	*P*<0.001	*P*<0.001
	≥30	Group 1 (n=84)	3.75 ±0.67	3.58 ±0.65	3.65 ±0.66	3.68 ±0.62	87.98 ±12.6
		Group 2 (n=59)	3.48 ±0.94	3.48** ±0.79	3.59 ±0.89	3.26 ±0.82	82.9* ±14.44
	Difference (p)		NS	NS	NS	*P*<0.01	*P*<0.05
	Active	Group 1 (n=51)	3.89 ±0.61	3.75 ±0.69	3.78 ±0.61	3.85 ±0.59	91.65 ±11.33
		Group 2 (n=125)	3.39 ±0.77	3.28 ±0.78	3.47 ±0.77	3.28 ±0.7	80.54 ±12.11
Physical activity	Difference (p)		*P*<0.001	*P*<0.001	*P*<0.01	*P*<0.001	*P*<0.001
	Non-active	Group 1 (n=163)	3.59** ±0.68	3.49* ±0.7	3.67 ±0.7	3.62* ±0.67	86.28* ±12.72
		Group 2 (n=143)	3.28 ±0.68	3.13 ±0.68	3.39 ±0.68	3.08* ±0.68	77.29* ±12.44
	Difference (*P*)		*P*<0.001	*P*<0.001	*P*<0.001	*P*<0.001	*P*<0.001

* *P*<0.05;

** *P*<0.01 differences between subgroups inside the groups

A considerable positive correlation between the age and levels of healthy behaviour of the subjects in the pilot group was observed. This correlation was not observed amongst pregnant women (Table 4). There were no correlations found between levels of healthy behaviour and BMI (in both groups) and between levels of healthy behaviour and week of pregnancy (Group 1).

**Table 4:** Correlations (r) between the age and level of health-related behaviour (NH – nutrition habits, PB – prophylactic behaviour, PA – positive attitude, HP – healthy practices, HRB – health-related behaviours)

***Group***	***NH***	***PB***	***PA***	***HP***	***HRB***
Group 1	0.167	0.150	0.102	0.135	0.181
Group 2	0.283**	0.314**	0.173	0.194*	0.342***

* *P*<0.05;

** *P*<0.01;

*** *P*<0.001

## Discussion

The majority of publications dealing with pregnant women analyse chronic pain of the spine and lower limbs (21–25). The application of a majority of physiotherapeutic treatments are contra-indicated for pregnant women, their pain is better reduced by virtue of massage and kinesiotherapy (9, 26–29). However, by providing some basic information about lumbar spine pain and its prevention, pregnant women were able to alter their lifestyle to better cope with the pain (13). Results could best be achieved in the treatment of the lower spine through proper instruction and correct posture training (23). Spending free time engaged in physically-active pursuits had on quicker recovery times after experiencing persistent back pain, confirming that physical exercise can help to considerably reduce pain amongst women (30). Pain hinders everyday functioning, housekeeping, looking after the children, and professional work alike. A lack of stability in the stomach muscles results in the accumulation of the trunk load in the lower part of the trunk, with static-dynamic equilibrium being disturbed. Through consistent physical exercise, pain levels are lessened during the course of exercise or soon thereafter (30).

An active lifestyle positively affects the realm of mental health as well, as exercise is recognized as a successful method of treating depression in the general population of adults. The positive effects of therapy through aerobics, when applied to people suffering from depression, suggests that regular exercise should be incorporated into the treatment of postpartum depression (31, 32). An examination of the one hundred sixty-one women who took part in the special M&B programme, which combined exercise for pregnant women with education, revealed a positive improvement with regards to the women’s moods and a reduction in the symptoms of depression (31). Aerobics done during pregnancy prevents many complications from arising. On one hand, maintaining a sedentary lifestyle during pregnancy increases the likelihood of the emergence of some enduring diseases. On the other hand, aerobics has a substantial impact on maintaining an appropriate body weight and musculoskeletal performance. In addition, a woman may experience fewer body-image issues after delivery (33). The positive effects of purposefully completing exercises and physical activity during pregnancy are evidenced by various research outcomes (9, 27, 29, 34). After having conceived, the majority of women decrease their proclivity to exercise and adopt a more passive lifestyle (4). This takes place for a variety of reasons: pain resulting from the biomechanical changes that occur during pregnancy, nausea and vomiting predominantly accompanying the first trimester, lack of knowledge about the possibility of doing aerobics, and fear of foetus damage is being chief among them (11). Pregnant women decide not to exercise in spite of the fact that research points outs the benefit achieved through doing aerobics during pregnancy, which include preventing overweight, arterial hypertension, and gestational diabetes, higher self-esteem, lower levels of stress and lower risks of suffering from postpartum depression, alleviation of disorders due to a woman’s body adapting to pregnancy (particularly lumbar spine pain and the swelling of the lower limbs), and an improvement in the quality and efficiency of sleep (9, 13, 16, 35). Moreover, active individuals take more care of other elements of living a healthy lifestyle. This interdependence applies not only to pregnant women, confirmed by research (36–39).

Pregnant women (examined within the frame of this research) reported higher levels of health-related behaviours than non-pregnant women did. During pregnancy, women evince greater interest in a healthier lifestyle and seek information related to the prevention of pain disorders, to adhering to a correct diet, etc. (40). Education every so often brings about improvements in the quality of life and shapes correct physical habits (19, 40).

In the face of increasing risks of disease in society, promoting a healthy lifestyle and physical activity, particularly among pregnant women, appears to be one of the crucial duties of medical professionals (doctors, midwives, physiotherapists), while research into the best practices and results of the aforementioned procedures should be continued on a larger scale.

## Conclusion

Pregnancy can predispose women to take a greater interest in the issues of their own health and leading a healthier lifestyle. Physical activity can have an impact on other health-related behaviours, which affirms both the educational function of health training and the transmitting of such desired behaviours to everyday life.

## Ethical considerations

Ethical issues (Including plagiarism, informed consent, misconduct, data fabrication and/or falsification, double publication and/or submission, redundancy, etc.) have been completely observed by the authors.
